# Plexin D1 emerges as a novel target in the development of neural lineage plasticity in treatment-resistant prostate cancer

**DOI:** 10.21203/rs.3.rs-4095949/v1

**Published:** 2024-03-27

**Authors:** Chengfei Liu, Bo Chen, Pengfei Xu, Joy Yang, Christopher Nip, Leyi Wang, Yuqiu Shen, Shu Ning, Yufeng Shang, Eva Corey, Allen C. Gao, Jason Gestwicki, Qiang Wei, Liangren Liu

**Affiliations:** UC Davis; University of California, Davis; UC Davis; University of California, Davis; University of California, Davis; University of California, Davis; UC Davis; University of California, Davis; University of Washington; UC Davis; University of California, San Francisco; West China Hospital of Sichuan University

**Keywords:** Prostate cancer, PlexinD1, enzalutamide resistance, neural lineage plasticity

## Abstract

Treatment-induced neuroendocrine prostate cancer (t-NEPC) often arises from adenocarcinoma via lineage plasticity in response to androgen receptor signaling inhibitors, such as enzalutamide. However, the specific regulators and targets involved in the transition to NEPC are not well understood. Plexin D1 (PLXND1) is a cellular receptor of the semaphorin (SEMA) family that plays important roles in modulating the cytoskeleton and cell adhesion. Here, we found that PLXND1 is highly expressed and positively correlated with neuroendocrine markers in patients with NEPC. High PLXND1 expression is associated with poorer prognosis in prostate cancer patients. Additionally, PLXND1 was upregulated and negatively regulated by androgen receptor signaling in enzalutamide-resistant cells. Knockdown or knockout of PLXND1 inhibit neural lineage pathways, suppressing NEPC cell proliferation, PDX tumor organoid viability, and xenograft tumor growth. Mechanistically, the chaperone protein HSP70 regulates PLXND1 protein stability through degradation, and inhibition of HSP70 decreases PLXND1 expression and NEPC organoid growth. In summary, our findings suggest that PLXND1 could be a new therapeutic target and molecular indicator for NEPC.

## Introduction

Prostate cancer is the most common cancer in males and the fifth leading cause of cancer-related deaths worldwide [1, 2]. The primary treatments for early stage and localized prostate cancer include radical prostatectomy and radical radiotherapy. In the United States, survival rates for localized, regional, and metastatic stages have been reported to be 100%, 96.1%, and 18.5%, respectively [3]. Androgen deprivation therapy (ADT) is a standard treatment for advanced prostate cancer. However, despite initial remission, the disease often progresses to castration-resistant prostate cancer (CRPC) [4].

Androgen receptor (AR) signaling inhibitors such as enzalutamide, abiraterone, and apalutamide have been developed for the treatment of CRPC [5, 6]. Unfortunately, patients undergoing this treatment often develop resistance via various mechanisms [7]. This resistance can lead to the emergence of neuroendocrine differentiation and its features [8]. Following AR-targeted therapy, prostate cancer cells undergo changes in their characteristics, resembling stem cell-like properties, due to tumor cell plasticity and molecular reprogramming. This transformation contributes to neuroendocrine trans-differentiation [9]. Neuroendocrine prostate cancer (NEPC) is a specific subset of CRPC that is resistant to AR signaling inhibitors. NEPC is characterized by low or absent AR expression, independence from AR signaling, and acquisition of a neuroendocrine phenotype [10]. *De novo* NEPC is rare, accounting for less than 2% of all primary prostate cancers [11]. Treatment-induced NEPC has been reported in 10–17% of patients with advanced therapy-resistant CRPC [12]. The survival rate of NEPC patients is poor, with a 5-year survival rate of only 10%[10, 13]. To maximize the survival benefits of patients with NEPC, a comprehensive understanding of the molecular mechanisms involved in NEPC development and progression is crucial.

Plexin D1 (PLXND1) is a member of the plexin family of transmembrane proteins that plays an essential role in axonal guidance and vascular patterning [14]. Normally, PLXND1 expression is low in adult tissues and is associated with a subset of activated fibroblasts and macrophages [15]. Studies have reported the upregulation of PLXND1 in various tumors, including pancreatic, melanoma, ovarian, colon, and prostate tumors [14, 16]. However, there are currently no reports available on the expression and function of PLXND1 in NEPC.

In this study, we found that enzalutamide not only activated neural lineage pathways but also induced the upregulation of PLXND1. The analysis indicated that PLXND1 was upregulated in NEPC samples and was associated with poor patient prognosis. Further investigation revealed that AR negatively regulates PLXND1 in enzalutamide-resistant prostate cancer cells. *In vitro* and *in vivo* studies have suggested that inhibition of PLXND1 could suppress NEPC. Additionally, the study identified that the HSP70 inhibition has the potential to degrade the protein expression of PLXND1. Overall, this study proposed that PLXND1 could serve as a prognostic marker and a potential target for treating NEPC.

## Materials and Methods

### Reagents and cell culture

H660, CWR22Rv1, HEK293, and HEK293T cells were obtained from the American Type Culture Collection (ATCC). CWR22Rv1 cells were cultured in RPMI 1640 supplemented with 10% fetal bovine serum (FBS), 100 units/ml penicillin, and 0.1 mg/ml streptomycin. H660 cells were cultured in RPMI 1640 supplemented with 5% FBS, 0.005 mg/ml insulin, 0.01 mg/ml transferrin, 30 nM sodium selenite, 10 nM hydrocortisone, 10 nM beta-estradiol, and 2 mM L-glutamine. HEK293 and HEK293T cells were cultured in DMEM supplemented with 10% FBS, 100 units/ml penicillin, and 0.1 mg/ml streptomycin. C4–2B (a kind gift from Dr. Leland Chung) cells were cultured in RPMI 1640 supplemented with 10% fetal bovine serum (FBS), 100 units/ml penicillin, and 0.1 mg/ml streptomycin.C4–2B-MDVR (C4–2B enzalutamide-resistant) cells were maintained in medium containing 20 μM enzalutamide [17]. C4–2B parental cells were passaged concurrently with the resistant cells as controls. All cells were maintained at 37°C in a humidified incubator with 5% CO2. Enzalutamide was obtained from Selleck Chemicals and was dissolved in DMSO. JG231, synthesized as previously described, was confirmed by 1H NMR and LC-MS/MS, with a purity exceeding 95%, as determined by HPLC.

### Plasmids and cell transfection

For small interfering RNA (siRNA) transfection, cells were plated at a density of 0.5 × 10^5 cells per well in 12-well plates or 2 × 10^5 cells per well in six-well plates. Transfection was performed with 20 nM siRNA targeting the PLXND1 sequence (IDT Catalog#420764235 and 420764238), HSP70 sequence (HSPA1B, Invitrogen Catalog# 262305 and 262306), or control siRNA (Invitrogen Catalog# 12935300), using Lipofectamine-iMAX (Invitrogen). The effect of siRNA-mediated gene silencing was assessed by qRT-PCR and western blot analysis 3–4 days post-transfection.

LentiCRISPR v2 plasmids for PLXND1 knockout were designed and constructed following a previously published protocol [18]. The oligonucleotides used for sgControl and sgPLXND1 plasmids were as follows: sgControl, 5′-CACCGGGGCGAGGAGCTGTTCACCG-3′ (forward) and 5′-AAACCGGTGAACAGCTCCTCGCCCC-3′ (reverse); sgPLXND1#1, 5′-CACCGGCGTCAACAACTACACAGCG-3′ (forward) and 5′-AAACCGCTGTGTAGTTGTTGACGCC-3′ (reverse); sgPLXND1#2, 5′-CACCGTCTTCCTGGGCACGGTCAAC – 3′ (forward) and 5′-AAACGTTGACCGTGCCCAGGAAGAC-3′ (reverse). Lentiviral particles were generated in HEK293T cells by co-transfection with the lentivirus vectors, psPAX2 and pMD2.G. The lentivirus-containing medium was collected and the target cells were subsequently infected.

### Real-time quantitative PCR

Total RNA was extracted using an RNeasy Mini Kit (Qiagen, Hilden, Germany). Subsequently, cDNA was synthesized according to the manufacturer’s instructions and used for real-time quantitative PCR (RT-qPCR) with ssoFast Eva Green Supermix (Bio-Rad) according to the manufacturer’s instructions. Each reaction was normalized to the co-amplification of GAPDH. The primer sequences for real-time PCR were as follows: PLXND1, 5′-GTATCGGCCGCAGATCATGG – 3′ (forward) and 5′-TCCTCCATCAAAGCCACCAC-3′ (reverse). AR-FL: 5′-AAGCCAGAGCTGTGCAGATGA-3′ (forward) and 5′-TGTCCTGCAGCCACTGGTTC-3′ (reverse). SYP: 5′-TCGTGTTCAAGGAGACAGGC-3′ (forward) and 5′-GGCTCATTGACCAGACTACA-3′ (reverse). ChgA, 5′-CAAGACCTCGCTCTCCAAGG-3′ (forward) and 5′-TGGCTGCTCTGGTTCTCAAG-3′ (reverse). ENO2: 5′-TCAAGGACAAGTATGGCAAGG-3′ (forward) and 5′-ACCGATCACCATCTTTTCCG-3′ (reverse). GAPDH: 5′-ATGAGTCCTTCCACGATACCA-3′ (forward) and 5′-GAAATCCCATCACCATCTTCC-3′ (reverse).

### Western blot analysis

Whole-cell protein extracts were separated by SDS-PAGE and transferred onto nitrocellulose membranes. Blocking was performed using 5% milk in PBS containing 0.1% Tween-20 for 1 h at room temperature. Subsequently, membranes were incubated overnight at 4 °C with primary antibodies, including HSP70 (1:1000 for WB, 1:200 for IP, Cell Signaling Technology Cat#4873, RRID:AB_2119694), GAPDH (1:1000, Cell Signaling Technology Cat#2118, RRID: AB_561053), Tubulin (T5168, 1:5000, Sigma-Aldrich), PLXND1 (1:1000, Cell Signaling Technology Cat#92470, RRID:AB_2800187), SYP (1:1000, ThermoFisher, Cat#PA5–16417, RRID:AB_10989504), CHGA (1:1000, Santa Cruz Biotechnology, Cat#393941, RRID:AB_2924720), NSE (1:1000, Santa Cruz Biotechnology, Cat#sc-271384, RRID:AB_10609119), CDK2 (1:1000, Cell Signaling Technology Cat#2546, RRID:AB_2276129), CDK4 (1:1000, Cell Signaling Technology Cat#12790, RRID:AB_2631166), Cyclin A (1:1000, Cell Signaling Technology Cat#4654, RRID:AB_10614011), Cyclin D1 (1:1000, Cell Signaling Technology Cat#2978, RRID:AB_2259616), Cyclin E (1:1000, Cell Signaling Technology Cat#4132, RRID:AB_2071197), Cleaved-Caspase 7 (1:1000, Cell Signaling Technology Cat#9491, RRID:AB_2068144), Cleaved-PARP1:1000, Cell Signaling Technology Cat#5625, RRID:AB_10699459). Following primary antibody incubation, the membranes were treated with secondary antibodies (1:5000 dilution, Promega Cat#W4021, RRID: AB_430834), and immunoreactive proteins were visualized using an enhanced chemiluminescence detection system (Millipore, Billerica, MA, USA).

### Co-immunoprecipitation assay

Equal amounts of cell lysates (1500 μg) were subjected to immunoprecipitation overnight using 1 μg of specific antibodies, such as HSP70, along with 100 μL of protein A/G agarose with continuous rotation. The immunoprecipitants were washed twice with 1 mL 10 mM HEPES (pH 7.9), 1mM EDTA, 150 mM NaCl, and 1% Nonidet P-40. The precipitated proteins were eluted with 60 μL SDS-PAGE sample buffer by boiling for 10 min. The eluted proteins were separated on a 6% SDS-PAGE gel, transferred to nitrocellulose membranes, and incubated with the specified antibodies.

### Cell growth assay

C4–2B-MDVR and CWR22Rv1 cells were plated in 12-well plates at a density of 1 × 10^4 cells/well in RPMI 1640 medium supplemented with 10% FBS and subjected to various treatments. H660 cells were seeded in 12-well plates at a density of 5 × 10^4 cells/well in the appropriate medium for H660 culture and were treated under various conditions. The overall cell count was used to calculate the percentage of surviving cells.

### Clonogenic assay

C4–2B-MDVR and CWR22Rv1 cells were seeded at a density of 1000 cells/well in 6-well plates and exposed to various treatments for 11 d. Subsequently, the colonies were washed with PBS and stained with 0.5% crystal violet/4% formaldehyde for 30 min, and the colony count was determined.

### RNA-seq data analysis

Total RNA from both control and PLXND1-knockdown C4–2B-MDVR and H660 cells was extracted using the RNeasy Mini Kit (Qiagen) and subjected to DNase digestion, according to the manufacturer’s instructions. Subsequently, RNA-seq libraries were generated using 1 μg of total RNA and the Illumina TruSeq RNA Sample kit. Paired-end mRNA-Seq libraries were constructed on an Illumina HiSeq 4000 platform with 2 × 150 cycles/bases (150bp, PE), resulting in approximately 30 million reads per sample. Data analysis involved a Top Hat-Cufflinks pipeline, with sequence read mapping and alignment performed using HISAT. The obtained StringTie data were then mapped and quantified for 27,044 unique genes/transcripts, with gene and transcript expression quantified as FPKM (Fragments Per Kilobase of transcript per million mapped reads).

### Gene set enrichment analysis (GSEA)

GSEA was performed using Java desktop software (http://software.broadinstitute.org/gsea/index.jsp) as described previously [19]. Significance was attributed to pathways showing enrichment with a normalized enrichment score (NES), a nominal p-value below 0.05, and an FDR q-value less than 0.25.

### PDX tumor xenografts and organoid culture

Experimental procedures involving animals were approved by the Institutional Animal Care and Use Committee of UC Davis and adhered to the ARRIVE guidelines, ethical regulations, and human endpoints (animal protocol number #22246). Male 6-week-old SCID mice were procured from Envigo and housed in an animal research facility at UC Davis. To assess the impact of PLXND1 on tumor development and growth, 4 × 10^6 CWR22Rv1 cells (sgControl, sgPLXND1#1, or sgPLXND1#2) were combined in a 1:1 ratio with Matrigel (Corning) for bilateral subcutaneous injection into NSG mice. Tumor size was measured every 3–4 days with calipers, and tumor volume was calculated as widtĥ2 × length × 0.52, starting one week after tumor inoculation.

For organoid culture, PDX tumor tissues were collected and cut into 2–4 mm^3^. Tumors were digested using collagenase IV (STEMCELL) and incubated at 37°C for 30 min until tumor cells were dispersed. Advanced DMEM (ADMEM) medium supplemented with 1× GlutaMAX (Gibco), 1M HEPES (Gibco), 100 u/ml penicillin, and 0.1 mg/ml streptomycin was added to the cell suspension and then filtered through 40 μm cell strainers to obtain a single-cell suspension. The cells were then centrifuged and resuspended in ADMEM complete medium containing GlutaMAX (Gibco), HEPES (Gibco), 100 u/ml penicillin, and 0.1 mg/ml streptomycin, B27 (Gibco), N-Acetylcysteine (Thermo Scientific), Human Recombinant EGF (Thermo Scientific), Recombinant FGF-10 (Invitrogen), A-83–01 (Tocris), SB202190 (Bioscience), Nicotinamide (Thermo Scientific), dihydrotestosterone (Sigma), PGE2 (Bioscience), Noggin (Thermo Scientific), and R-spondin (R&D Systems). Tumor cells were seeded in a 96-well plate with Matrigel diluted in a 1:3 ratio of ADMEM complete medium and incubated at 37°C for 15 min to solidify the matrigel complex. Next, ADMEM complete medium mixed with JG231, packaged siRNA, or packaged LentiCRISPR v2 plasmid was added to each well. The viability of the organoids was analyzed using the CellTiter-Glo Luminescent assay (Promega) and visualized by immunofluorescence using the LIVE/DEAD^®^ Viability/Cytotoxicity Assay Kit (Thermo Scientific) according to the manufacturer’s protocol.

### Immunohistochemistry

Tumors were fixed in formalin and paraffin-embedded tissue blocks were dewaxed, rehydrated, and blocked for endogenous peroxidase activity. Antigen retrieval was performed in sodium citrate buffer (0.01 mol per Litter, pH 6.0) in a microwave oven at 1000 W for 3 min and then at 200 W for 20 min. Non-specific antibody binding was blocked by incubation with 10% fetal bovine serum in PBS for 30 min at room temperature. Slides were then incubated with anti-PLXND1 ( 1:100, CST; or 1:200, Zen-bio) at 4°C overnight. The slides were then washed and incubated with biotin-conjugated secondary antibodies for 30 min, followed by incubation with avidin DH-biotinylated horseradish peroxidase complex for 30 min (Vectastain ABC Elite Kit, Vector Laboratories). The sections were developed using a diaminobenzidine substrate kit (Vector Laboratories) and counterstained with hematoxylin. Nuclear staining of cells was performed and the cells were counted in five different vision fields. Images were taken using an Olympus BX51 microscope equipped with a DP72 camera.

### Prostate adenocarcinoma and NEPC patient tissue samples

Primary prostate adenocarcinoma tissue microarray (n = 46) and NEPC samples (n = 3) were obtained from the Department of Urology at the West China Hospital, Sichuan University. Pathologists at the Department of Pathology, West China Hospital, Sichuan University confirmed the pathological types of prostate adenocarcinoma or NEPC. This study was approved by the Institutional Ethics Review Board of West China Hospital (No. 2017 – 324), and written informed consent was obtained from all patients. PLXND1 staining without awareness of patient clinical information by combining the area percent of positive cells from 1 to 4 (1 = 0%–25%, 2 = 26%–50%, 3 = 51%–75%, and 4 = 76%–100%) and the relative intensity of the staining gray level from 1 to 4 (1 = negative, 2 = low, 3 = medium, and 4 = high). All statistical significance was obtained using a z-score calculator for two population proportions (https://www.socscistatistics.com/tests/ztest/) between a group with scores of 1 and 2(negative to low intensity) and a group with scores of 3 and 4 (medium to high intensity).

### Datasets and patients’ cohort

Tumor sample information and corresponding clinical characteristics from various cohorts, including the Beltran cohort for castration-resistant neuroendocrine prostate cancer [20], Stand Up 2 Cancer/Prostate Cancer Foundation-funded West Coast Prostate Cancer Dream Team [21], Abida-Wassim cohort for metastatic castration-resistant prostate cancer [22], MSK Science 2022 (https://www.cbioportal.org/study/summary?id=prad_organoids_msk_2022), and The Cancer Genome Atlas Research Network [23], were obtained from the cBioPortal for cancer genomics (https://www.cbioportal.org/). Other RNA-seq raw data were accessible to the Gene Expression Omnibus (GEO) under accession numbers GSE160393, GSE126078, GSE21032, GSE66187, GSE215653, GSE151083, and GSE52169.

### Statistical analysis

Statistical analyses were performed using GraphPad Prism 9.0 (RRID:SCR_002798). Raw data were summarized by means, standard deviations (SD), and graphical summaries and then transformed, if necessary, to achieve normality. The sample size was determined based on the power to detect significant differences *(p < 0.05)*. No samples or data points were excluded from the analysis. The experiments and data processing were not blinded. Data are presented as the mean ± SD from three independent experiments. Differences between individual groups were analyzed using a two-tailed Student’s t-test for single comparisons or one-way analysis of variance (ANOVA), followed by the Scheffé procedure for multiple group comparisons. In the tumor growth experiments, the size of the tumor at sacrifice served as the primary response measure. Tumor growth across groups was analyzed using analysis of variance. P < 0.05 was considered statistically significant (*P < 0.05, **P < 0.01, ***P < 0.001, ****P < 0.0001, ns = non-significant).

### Data availability statement

The data obtained in this study are available upon reasonable request from the corresponding authors.

## Results

### PLXND1 is upregulated in NEPC and prostate cancer cells showing neural lineage plasticity

**Figure 1:**
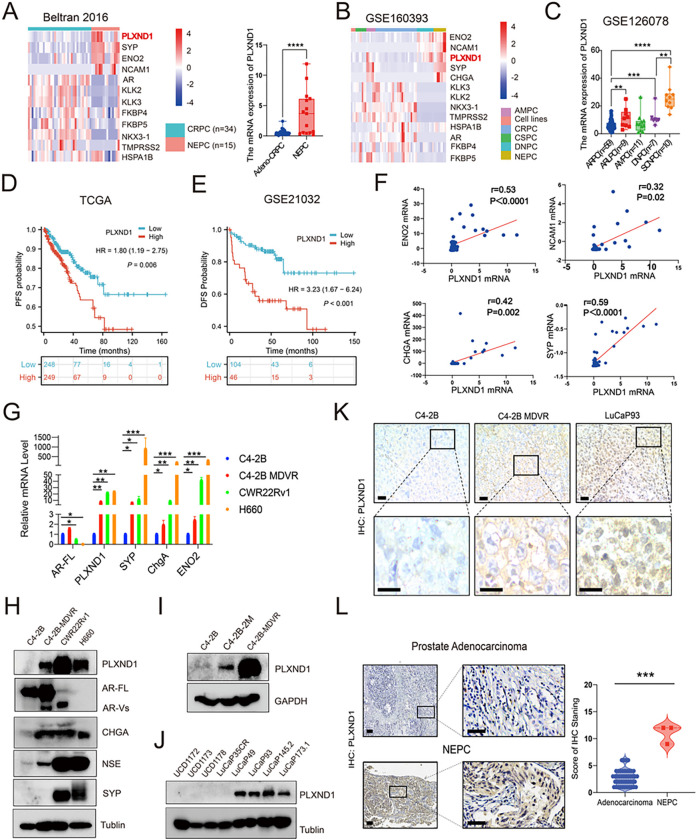
PLXND1 is upregulated in NEPC and prostate cancer cells showing neural lineage plasticity. **A-B.**Heatmap to show the expression levels of PLXND1 and other genes in cell lines, CRPC, CSPC, DNPC, AMPC, and NEPC from the Beltran 2016 and GSE160393 datasets respectively. **C.** Comparisons of PLXND1 mRNA levels in ARPC, ARLPC, AMPC, DNPC, and SCNPC. **D-E.** Kaplan Meier curve to show the correlation between PLXND1 expression levels and patients’ prognosis form the TCGA and GSE21032 datasets respectively. **F.** Pearson correlation analysis of PLXND1 versus ENO2, NCAM1, CHGA, and SYP mRNA in Beltran 2016 cohort from cBioPortal database. **G.** mRNA expression of AR-FL, PLXND1, SYP, ChgA, and ENO2 in C4–2B, C4–2B-MDVR, CWR22Rv1, and H660 cells. **H.**Western blot of PLXND1 and other indicated proteins in C4–2B, C4–2B-MDVR, CWR22Rv1, and H660 cells. **I.** Western blot of PLXND1 proteins in C4–2B, C4–2B-ENZA 2 months, and C4–2B-MDVR cells. **J.** Western blot of PLXND1 proteins in CRPC PDX tumors (UCD1172, UCD1173, UCD1178 and LuCaP35CR) and NEPC PDX tumors (LuCaP49, LuCaP93, LuCaP145.2 and LuCaP173.1). **K.** Representative PLXND1 IHC staining in prostate tumors (C4–2B, C4–2B-MDVR, and LuCaP93). Scale bar represents 20 microns (upper) and 10 microns (lower), respectively. **L.** Representative PLXND1 IHC staining and quantification in patients’ samples (prostate adenocarcinoma versus NEPC). Scale bar represents 100 microns (left) and 50 microns (right), respectively. **P < 0.01, ***P < 0.001, ****P < 0.0001.

Analysis of publicly available RNA-seq datasets revealed upregulated mRNA levels of PLXND1 and neuroendocrine feature genes (CHGA, NCAM1, SYP, and ENO2), along with downregulated AR and its targeting genes (KLK2, KLK3, NKX3–1, TMPRSS2, FKBP4, and FKBP5) in NEPC tumors compared to CRPC (Beltran 2016, GSE160393) (Fig. 1A, **Fig.S1D**) [20], which was observed in both AMPC (amphicrine expression of both AR and neuroendocrine markers) and NEPC, compared to CRPC, castration-sensitive prostate cancer (CSPC), and DNPC (double-negative expression of AR and neuroendocrine markers) (GSE160393, GSE126078) (Fig. 1B–1C) [24, 25]. Additionally, HuPSA (Human Prostate Single cell Atlas) data analysis demonstrated higher PLXND1 mRNA levels in NEPC patients by considering the heterogeneous populations of prostate cancer cells (**Fig.S1A**). To investigate whether enzalutamide treatment affects PLXND1 expression, data analysis showed that PLXND1 was upregulated in enzalutamide-treated prostate cancer cells compared to the DMSO treatment group (GSE215653) (**Fig.S1B**) [26]. Furthermore, our RNA-seq data revealed upregulation of both PLXND1 and neuroendocrine markers, such as ENO2, CHGA, NCAM1, and SYP, in C4–2B-MDVR cells compared with parental C4–2B cells (**Fig.S1E**). GSEA analysis indicated a significant enrichment of neural lineage pathways in enzalutamide-treated prostate cancer cells (GSE215653 and GSE 151083) (**Fig.S1F-S1G**) [26, 27], including pathways related to synapse assembly, synaptic membrane, neuron projection terminus, synaptic transmission glutamatergic, neurotransmitter receptor activity, and distal axons. To assess the association between PLXND1 expression levels and prostate cancer patient prognosis, Kaplan-Meier survival analysis demonstrated that patients with higher PLXND1 expression had inferior survival rates compared to those with lower PLXND1 expression (TCGA: HR = 1.80 (1.19–2.75), P = 0.006; GSE21032: HR = 3.23 (1.67–6.24), P < 0.001) [22, 28]. Correlation analysis revealed a positive correlation between PLXND1 expression and neuroendocrine features (Beltran 2016: CHGA (r = 0.42, P = 0.002), ENO2 (r = 0.53, P < 0.0001), SYP (r = 0.59, P < 0.0001), and NCAM1 (r = 0.32, P = 0.02; GSE126078: CHGA (r = 0.59, P < 0.0001), ENO2 (r = 0.60, P < 0.0001), SYP (r = 0.43, P < 0.0001), and NCAM1 (r = 0.55, P < 0.0001)) (Fig. 1F **and Fig.S1H**) [20, 25]. We measured PLXND1 expression and associated neuroendocrine characteristics across different human prostate cancer cell lines, patient-derived xenograft (PDX) tumors, and prostate cancer patient samples. The results showed that PLXND1 was negative in C4–2B cells but was highly expressed in NE-like or NEPC cell lines (C4–2B-MDVR, CWR22Rv1, and H660) (Fig. 1G–1H). Further investigation revealed upregulated PLXND1 expression in C4–2B-2M (enzalutamide treatment for 2 months) and C4–2B-MDVR cells compared to that in parental C4–2B cells (Fig. 1I). Additionally, PLXND1 was more highly expressed in NEPC PDX tumors (LuCaP 49, LuCaP 93, LuCaP 145.2, and LuCaP 173.1) than in CRPC PDX tumors (UCD1172, UCD1173, UCD1178, and LuCaP35CR) (Fig. 1J). Immunohistochemistry (IHC) staining confirmed high PLXND1 expression in NEPC tumors (C4–2B-MDVR and LuCaP 93) compared to that in C4–2B tumors (Fig. 1K). Importantly, we screened 46 CRPC and three NEPC patient samples and found that PLXND1 staining was significantly increased in the NEPC patient samples (Fig. 1L). In summary, the data suggest that PLXND1 is upregulated in NEPC and cells gaining neural lineage plasticity features.

### AR signaling negatively regulates the expression of PLXND1 in enzalutamide-resistant prostate cancer.

**Figure 2:**
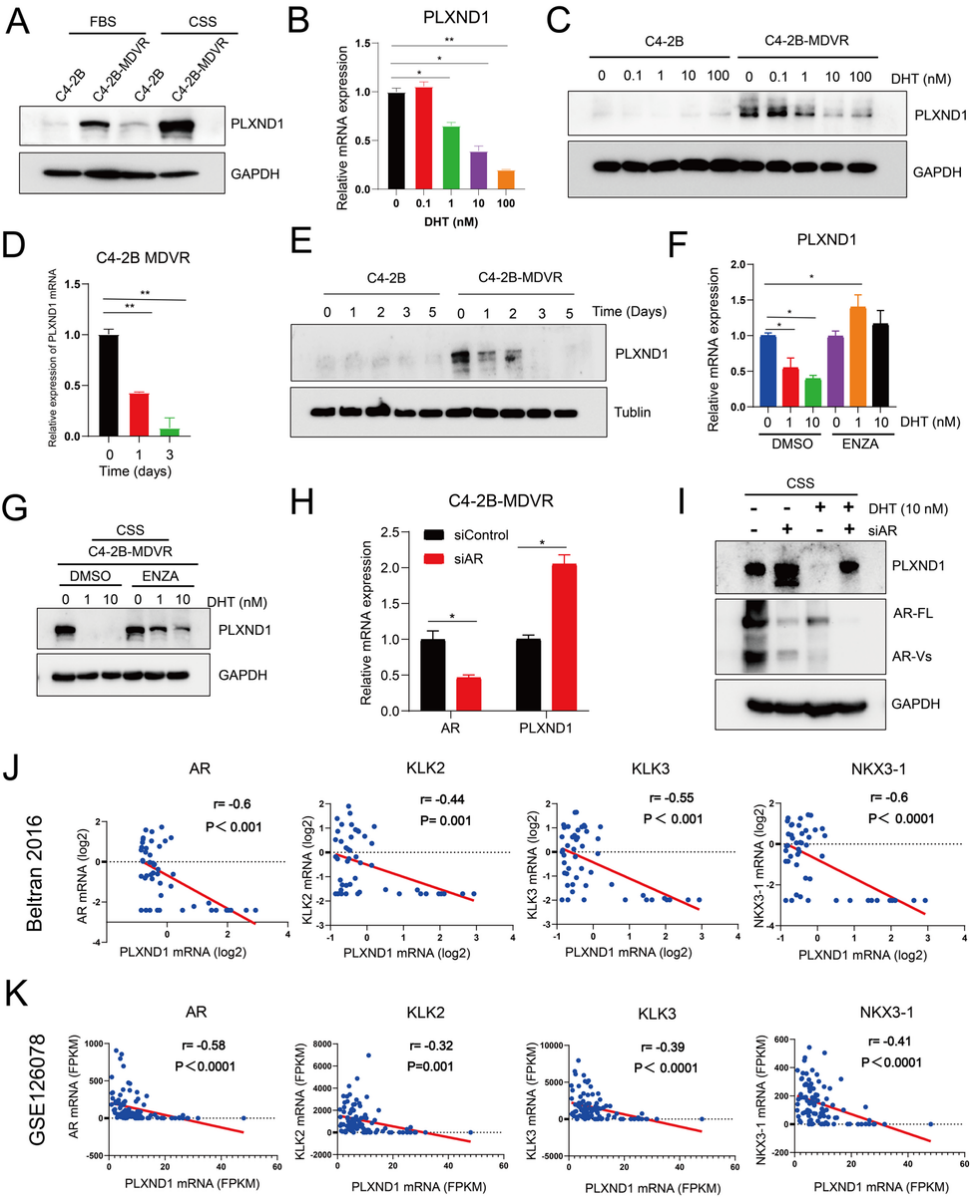
AR signaling negatively regulates the expression of PLXND1 in enzalutamide-resistant prostate cancer. **A.** Western blot showing PLXND1 in parental C4–2B and C4–2B-MDVR cells cultured in FBS or CSS media for 5 days respectively. **B.** RT-PCR was used to test the mRNA levels of PLXND1 in C4–2B and C4–2B-MDVR cells treated with different doses of DHT (0, 0.1, 1, 10, and 100 nM) for 3 days in CSS media. **C.** Western blot was used to test the protein levels of PLXND1 in C4–2B and C4–2B-MDVR cells treated with different doses of DHT (0, 0.1, 1, 10, and 100 nM) for 3 days in CSS media. **D.**RT-PCR was used to test the mRNA levels of PLXND1 in C4–2B-MDVR cells treated with 10 nM DHT for different timepoints (0, 1, and 3 days) in CSS media. **E.** Western blot was used to test the protein levels of PLXND1 in C4–2B-MDVR cells treated with 10 nM DHT for different timepoints (0, 1, 2, 3, and 5 days) in CSS media. **F-G.** C4–2B-MDVR cells were treated with DHT (0, 1, and 10 nM) in the absence or presence of enzalutamide (20 μM) for 3 days. RT-PCR was used to test the mRNA levels of PLXND1, and Western blot was used to test the protein levels of PLXND1, respectively. **H.** C4–2B-MDVR cells were transfected with siControl and siAR siRNAs for 3 days in CSS media. Then, the mRNA expression levels of AR and PLXND1 were determined by RT-PCR. **I.** Total lysates from C4–2B-MDVR cells transfected with or without siAR for 3 days and treated with or without 10 nM DHT for 2 days in CSS media were tested for AR-FL and PLXND1 expression by Western blot. **J-K.**Pearson correlation analysis of PLXND1 versus AR, KLK2, KLK3, and NKX3–1 mRNA in Beltran 2016 cohort and GSE126078 from dataset, respectively. * p<0.05, **P < 0.01.

To examine whether AR signaling regulates the expression of PLXND1, PLXND1 protein expression was determined in C4–2B-MDVR cells maintained in charcoal-stripped fetal bovine serum (CS-FBS) and regular FBS conditions. PLXND1 expression was significantly upregulated in CS-FBS compared to FBS (Fig. 2A). Both RNA-seq and GEO data (GSE52169) revealed that dihydrotestosterone (DHT) treatment significantly downregulated the mRNA levels of PLXND1 in prostate cancer (**Fig.S2A-S2B**) [29]. Further investigation confirmed that DHT treatment significantly suppressed the mRNA and protein expression of PLXND1 in a dose- and time-dependent manner in C4–2B-MDVR cells (Fig. 2B–2E). Additionally, DHT and enzalutamide combination treatment showed that enzalutamide could rescue both PLXND1 mRNA and protein expression inhibited by DHT in C4–2B-MDVR cells (Fig. 2F–2G). To determine whether the AR protein regulates PLXND1 expression, full-length AR (AR-FL) was knocked down with siRNA in C4–2B-MDVR cells. As shown in Fig. 2H–2I and **Fig.S2C**, AR-FL knockdown significantly increased the expression of PLXND1 mRNA and protein. Furthermore, DHT significantly reduced PLXND1 expression, and AR-FL knockdown blocked the inhibitory effect of DHT on PLXND1 expression (Fig. 2I, **Fig.S2C**). This was also confirmed by analyzing prostate cancer patient cohorts (Fig. 2J–2K, **Fig.S2D-S2E**). AR and its downstream targeting genes, such as KLK2, KLK3, and NKX3–1, were negatively correlated with PLXND1 in Beltran 2016, GSE126078, SU2C/PCF, and MSK 2022 databases [20, 21, 25]. In summary, these data suggest that AR signaling negatively regulates PLXND1 expression in enzalutamide-resistant prostate cancer cells.

### Knockdown of PLXND1 represses the cell proliferation and improves enzalutamide treatment.

**Figure 3:**
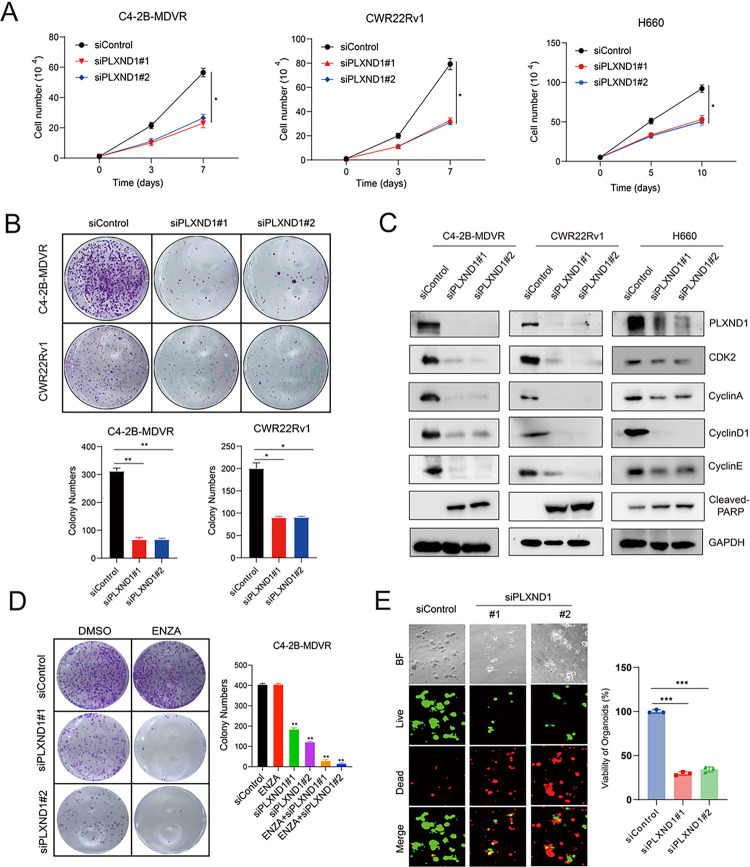
Knockdown of PLXND1 represses the cell proliferation and improves enzalutamide treatment. **A.** C4–2B-MDVR (1*10^4 cells/well), CWR22Rv1 (1*10^4 cells/well), and H660 (5*10^4 cells/well) cells were plated in 12-well plates and transfected with siControl or siPLXND1 siRNAs, respectively. Cell proliferation viability was determined by cell counting. **B.** C4–2B-MDVR and CWR22Rv1 cells were plated in 6-well plates, 1000 cells/well, and colony formation viability was determined by counting after transfected with siControl or siPLXND1 siRNAs for 10 days. **C.**C4–2B-MDVR, CWR22Rv1, and H660 cells after transfected with siControl or siPLXND1 siRNAs for 5 days. Then, whole cell lysates of C4–2B-MDVR, CWR22Rv1, and H660 cells were harvested for testing PLXND1 and other proteins expression levels. **D.** C4–2B-MDVR cells were plated in 6-well plates, 1000 cells/well, and colony formation was determined by counting after transfected with siControl or siPLXND1 siRNAs with or without 20 μM enzalutamide for 10 days. **E.** Cells from H660 PDX tumors were plated in 96-well plates, 1*10^4 cells/well, and transfected with siControl or siPLXND1 siRNAs for 10 days. Organoids viability was assayed by CellTiter-Glo Luminescent assay and the live-and-dead cells were visualized by immunofluorescence. * p<0.05, **P<0.01, ***P < 0.001.

To evaluate whether PLXND1 plays a role in regulating the aggressive characteristics of neuroendocrine prostate cancer (NEPC) cells, we employed two distinct siRNAs to knockdown PLXND1 in C4–2B-MDVR, CWR22Rv1, and H660 cells, all of which exhibit neuroendocrine traits [30–32]. The knockdown effect was validated using RT-qPCR (**Fig.S3A**). Subsequently, we observed that PLXND1 knockdown suppressed cell proliferation and colony formation in C4–2B-MDVR cells compared with the control groups. Similar inhibitory effects were observed in CWR22Rv1 and H660 cells (Fig. 3A–3B). Additionally, PLXND1 knockdown led to reduced expression of CDK2, CyclinA, CyclinD1, and CyclinE, along with increased expression of cleaved-PARP in C4–2B-MDVR, CWR22Rv1, and H660 cells (Fig. 3C). Importantly, we found that PLXND1 knockdown significantly improved enzalutamide treatment of C4–2B MDVR and CWR22Rv1 cells (Fig. 3D **and Fig.S3B**). Moreover, we assessed the effect of PLXND1 knockdown in an H660 organoid model. The results demonstrated that Silencing PLXND1 expression via siRNA significantly inhibited the viability and growth of H660 organoids (Fig. 3E). In summary, these findings suggest that PLXND1 could potentially serve as a therapeutic target for NEPC, given its involvement in regulating key cellular processes and aggressive behavior of NEPC cells.

### Knockdown of PLXND1 decreases the neuroendocrine traits.

**Figure 4:**
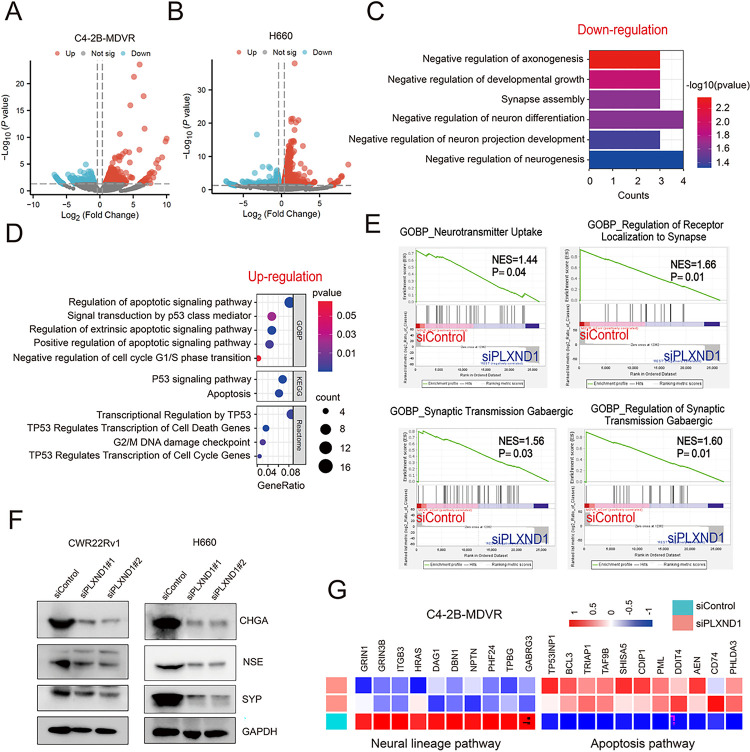
Knockdown of PLXND1 decreases the neuroendocrine traits. **A.** Volcano plot showing the differentiated expressed genes in C4–2B-MDVR cells with PLXND1 knockdown. **B.**Volcano plot showing the differentiated expressed genes in H660 cells with PLXND1 knockdown. **C.** Pathways down-regulated in PLXND1 knockdown C4–2B-MDVR. **D.** GO analysis showing pathways up-regulated in PLXND1 knockdown C4–2B-MDVR cells . **E.** Enrichment plots of GSEA analyses for the neural lineage pathways in siPLXND1 group compared with siControl group in C4–2B-MDVR cells. **F.** Western blot showing CHGA, NSE, and SYP proteins in CWR22Rv1 and H660 cells transfected with siControl or siPLXND1 siRNAs for 5 days. **G.** Heatmap clustering the down-regulated genes and up-regulated genes in C4–2B-MDVR cells, respectively.

To evaluate the impact of PLXND1 knockdown on neuroendocrine traits and associated pathways, we conducted RNA-seq analysis of C4–2B-MDVR and H660 cells with silenced PLXND1. The results revealed that 287 genes were significantly downregulated and 349 genes were significantly upregulated in C4–2B-MDVR cells (Fig. 4A). Similarly, in H660 cells, 431 genes were significantly downregulated and 1008 genes were significantly upregulated (Fig. 4B). Subsequent analysis showed that six neural lineage pathways were downregulated in C4–2B-MDVR cells with PLXND1 knockdown compared to the control, including the negative regulation of axonogenesis, developmental growth, synapse assembly, neuron differentiation, neuron projection development, and neurogenesis (Fig. 4C). GO analysis also revealed the downregulation of neural lineage pathways in H660 cells transfected with siPLXND1 (**Fig.S4A**). Further GOBP analysis indicated that five pathways were upregulated in C4–2B-MDVR cells with PLXND1 knockdown compared to the control, including regulation of the apoptotic signaling pathway, signal transduction by P53 class mediator, regulation of the extrinsic apoptotic signaling pathway, positive regulation of the apoptotic signaling pathway, and negative regulation of the cell cycle G1/S phase transition. KEGG analysis identified upregulation of the P53 signaling pathway and apoptosis in C4–2B-MDVR cells with PLXND1 knockdown compared with the control. Reactome analysis further revealed that four pathways regulating the cell cycle and apoptosis were significantly upregulated in C4–2B-MDVR cells with PLXND1 knockdown (Fig. 4D). GSEA analysis demonstrated that four neural lineage pathways were downregulated in C4–2B-MDVR cells with PLXND1 knockdown, including neurotransmitter uptake, regulation of receptor localization to synapses, synaptic transmission GABAergic, and regulation of GABAergic synaptic transmission (Fig. 4E). Apoptosis pathways were also upregulated in H660 cells transfected with siPLXND1 (**Fig.S4B**). Additionally, PLXND1 knockdown downregulated the protein expression of neuroendocrine markers, including CHGA, NSE, and SYP (Fig. 4F). Finally, heatmap analysis illustrated the downregulation of neural lineage pathway genes and the upregulation of apoptosis pathway genes in C4–2B-MDVR cells transfected with siPLXND1 (Fig. 4G). In summary, our data suggest that PLXND1 knockdown decreases neuroendocrine traits of NEPC cells.

### Knockout of PLXND1 by CRISPR/Cas9 inhibits the NEPC cells *in vitro*, organoids viability, and tumor growth *in vivo*.

**Figure 5:**
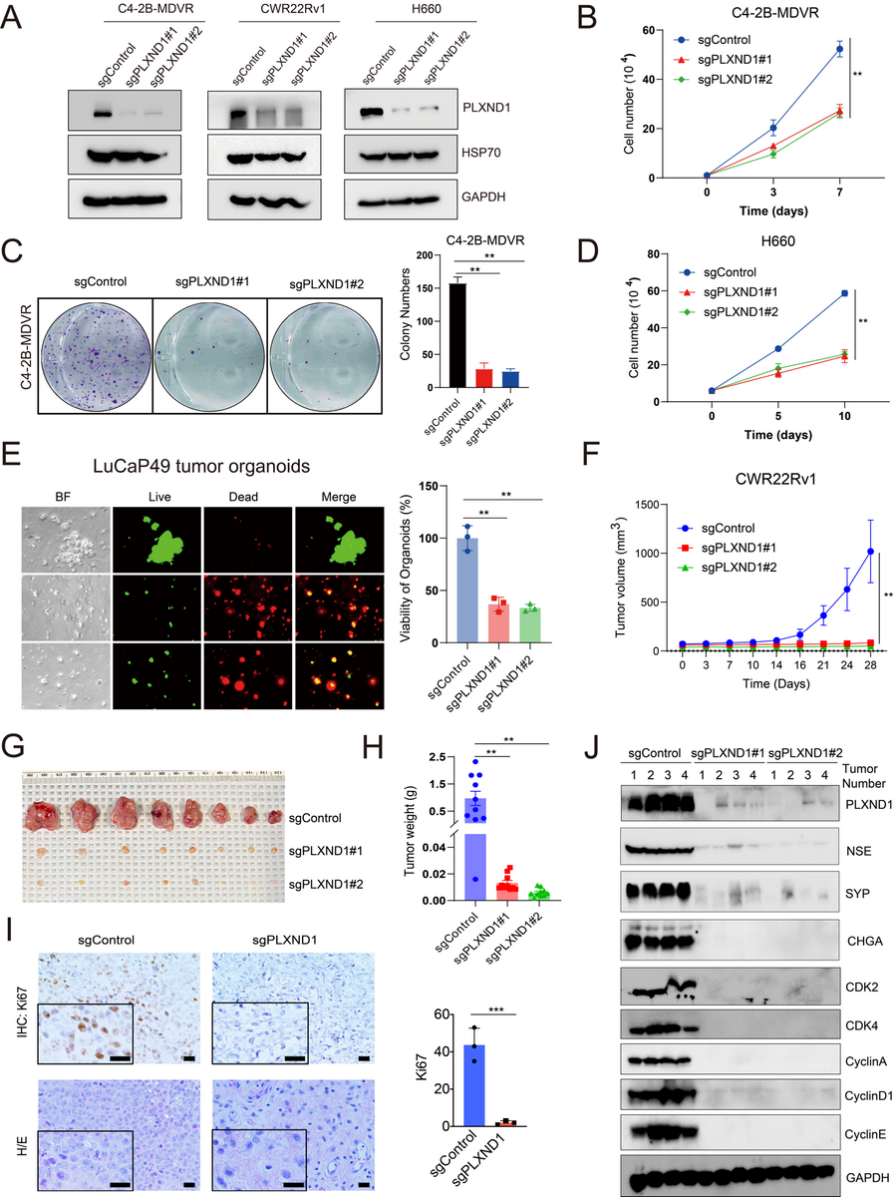
Knockout of PLXND1 by CRISPR/Cas9 inhibits the NEPC cells *in vitro*,organoids viability, and tumor growth *in vivo*. **A.** Western blot was used to confirm the PLXND1 knockout effect in C4–2B-MDVR, CWR22Rv1, and H660 cells transfected with sgControl or sgPLXND1 plasmids for 5 days, respectively. **B.** C4–2B-MDVR (1*10^4 cells/well) cells were plated in 12-well plates and transfected with sgControl or sgPLXND1 plasmids for 0, 3, and 7 days, respectively. Cell proliferation was determined by cell counting. **C.**C4–2B-MDVR cells were plated in 6-well plates, 1000 cells/well, and colony formation was determined by counting after transfected with sgControl or sgPLXND1 plasmids for 10 days, respectively. **D.** H660 (5*10^4 cells/well) cells were plated in 12-well plates and transfected with sgControl or sgPLXND1 plasmids for 0, 5, and 10 days, respectively. Cell viability was determined by cell counting. **E.** Cells from LuCaP49 PDX tumors were plated in 96-well plates, 1*10^4 cells/well, and transfected with sgControl or sgPLXND1 plasmids for 10 days. Organoids viability was assayed by CellTiter-Glo Luminescent assay and the live-and-dead cells were visualized by immunofluorescence. **F.** CWR22Rv1 tumors picture in sgControl, sgPLXND1#1, and sgPLXND1#2 group, respectively. **G-H.**CWR22Rv1 tumors growth curves and tumors weight in sgControl, sgPLXND1#1, and sgPLXND1#2 group, respectively. **I.** HE and IHC staining of Ki67 in sgControl and sgPLXND1 group were performed for CWR22Rv1 tumors. Scale bar represents 20 microns. **J.** Protein levels of PLXND1, NSE, CHGA, SYP, CDK2, CDK4, CyclinA, CyclinD1, and CyclinE in CWR22Rv1 tumors after PLXND1 knockout, using GAPDH as internal control, as determined by Western Blot. **P < 0.01, ***P < 0.001.

Subsequently, LentiCRISPR v2 plasmids were used to knock out PLXND1 in the C4–2B-MDVR, CWR22Rv1, and H660 cells. The knockout effect was validated by western blotting in C4–2B-MDVR, CWR22Rv1, and H660 cells (Fig. 5A). The results indicated that PLXND1 knockout suppressed proliferation and colony formation in C4–2B-MDVR cells compared to the control (Fig. 5B–5C), as well as in CWR22Rv1 (**Fig.S5A-S5B**) and H660 cells (Fig. 5D). Moreover, we assessed the effects of PLXND1 knockout in LuCaP49 and LuCaP93 organoid models and demonstrated that CRISPR/Cas9-mediated PLXND1 knockout significantly inhibited the viability and growth of LuCaP49 and LuCaP93 organoids (Fig. 5E, **Fig.S5C**). To determine whether PLXND1 knockout inhibited NEPC tumor growth *in vivo*, CWR22Rv1 cells, including sgControl, sgPLXND1#1, and sgPLXND1#2, were injected to establish xenografts. PLXND1 knockout significantly inhibited tumor growth in CWR22Rv1 xenografts (Fig. 5F–5H). IHC staining indicated that Ki67 expression was also suppressed in the sgPLXND1 group compared with that in the sgControl group (Fig. 5I). Furthermore, compared with the sgControl group, CDK2 and CDK4, along with their related cyclins, such as CyclinA, CyclinD1, and CyclinE, were significantly suppressed in the sgPLXND1 groups (Fig. 5J). In summary, these data confirm that PLXND1 affects NEPC proliferation and could serve as a potential therapeutic target for NEPC.

### HSP70 inhibition affects the protein stability of PLXND1.

**Figure 6:**
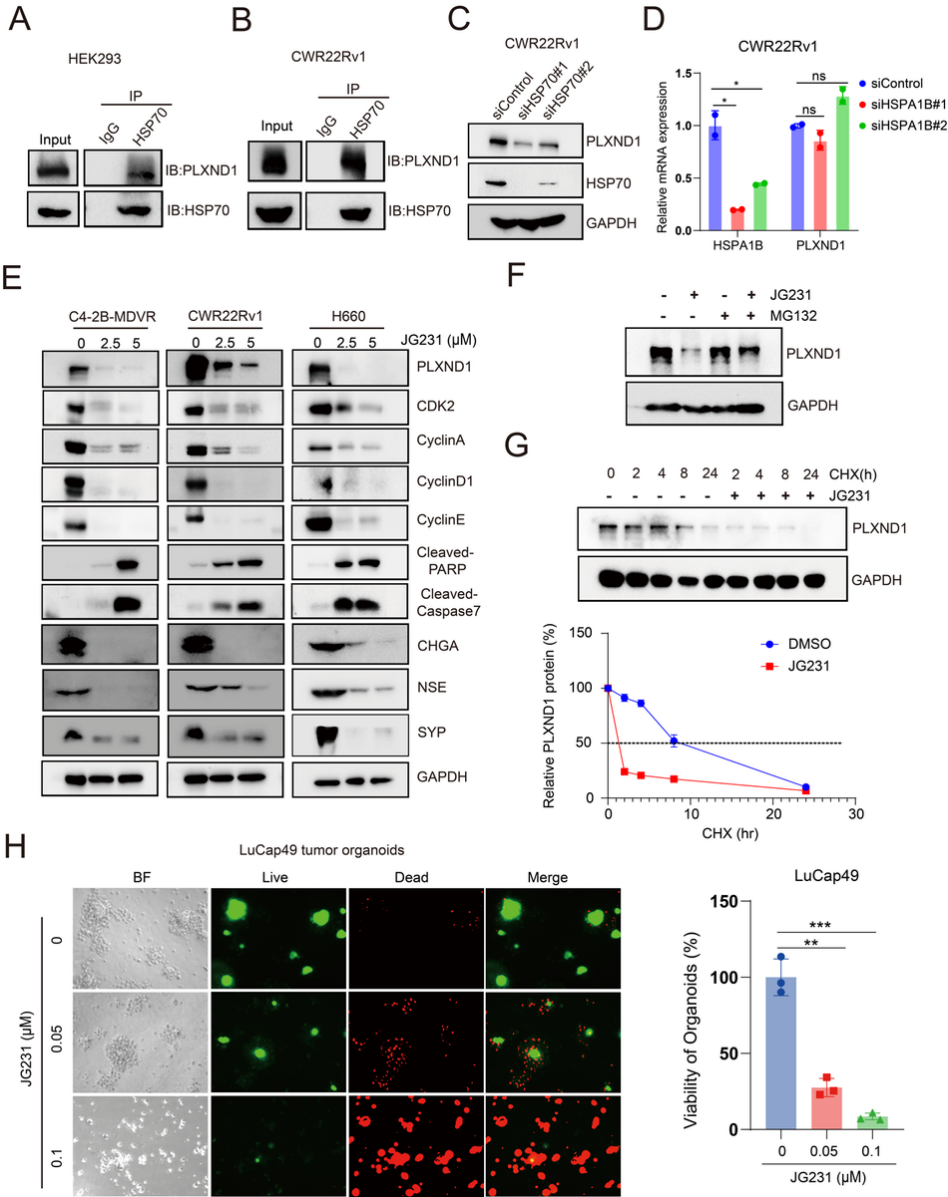
HSP70 inhibition affects the protein stability of PLXND1. **A-B.** Co-IP assays testing the correlation between PLXND1 and HSP70 in HEK293 and CWR22Rv1 cells, respectively. **C.** Western blot testing the proteins of PLXND1 and HSP70 in CWR22Rv1 cells transfected with siControl or siHSP70 siRNAs for 5 days. **D.** RT-PCR testing the mRNA of PLXND1 and HSPA1B in CWR22Rv1 cells transfected with siControl or siHSP70 siRNAs for 5 days. **E.** Western blot testing the proteins of PLXND1 and other proteins in C4–2B-MDVR, CWR22Rv1, and H660 cells treated with JG231 (0, 2.5, 5μM) for 24 hours. **F.** CWR22Rv1 cells were treated with or without JG231 (2.5 μM) for 24 hours in the absence or presence of the proteosome inhibitor, MG132 (50 μM) for 8 hours, and the protein expression of PLXND1 were analyzed by western blotting. **G.**CWR22Rv1 cells were treated with 50 μg/mL cycloheximide in the absence or presence of 5 μM of JG231. Total cell lysates were collected 0, 2, 4, 8, and 24 hours after treatment. The levels of PLXND1 were analyzed by western blotting to calculate the half-life of PLXND1. **H.** Organoids from LuCaP49 PDX tumors were treated with JG231(0.05 and 0.1 μM) for 9 days. Organoids viability was assayed by CellTiter-Glo Luminescent assay and the live-and-dead cells were visualized by immunofluorescence. (*P < 0.05, **P < 0.01, ***P < 0.001, ns = non-significant.

For a protein to become functional, it requires correct folding and assembly, a process that relies on the chaperone HSP70. To determine whether HSP70 binds to PLXND1 and regulates PLXND1 expression, we conducted co-IP assays in HEK293 and CWR22Rv1 cells. The results indicated that HSP70 binds to PLXND1 in both HEK293 and CWR22Rv1 cells (Fig. 6A–6B). Subsequently, we knocked down HSP70 in CWR22Rv1 cells using siRNA, and RT-PCR and western blotting were employed to validate the knockdown efficacy (Fig. 6C–6D). The findings revealed that HSP70 knockdown downregulated the protein expression of PLXND1 without affecting its mRNA expression of PLXND1 (Fig. 6C–6D). Using the HSP70 allosteric inhibitor JG231, we found that JG231 treatment significantly reduced the expression of PLXND1, CDK2, CyclinA, CyclinD1, CyclinE, and neuroendocrine markers (CHGA, NSE, and SYP) and increased the expression of cleaved-PARP and cleaved-Caspase7 in C4–2B-MDVR, CWR22Rv1, and H660 cells (Fig. 6E, **Fig.S6A**). To determine whether the decrease in PLXND1 protein expression induced by JG231 treatment was mediated through the proteasome pathway, we added the proteasome inhibitor MG132 to CWR22Rv1 cells. Although JG231 reduced PLXND1 protein expression, the addition of MG132 blunted the effects of JG231 (Fig. 6F). Furthermore, we investigated whether JG231 affects PLXND1 protein stability in CWR22Rv1 cells using the cycloheximide (CHX) chase assay and found that JG231 treatment significantly shortened the half-life of PLXND1 (Fig. 2G). Notably, JG231 treatment inhibited the growth of LuCaP49 and LuCaP93 NEPC organoids in a dose-dependent manner (Fig. 6H, **Fig.S6B**). Collectively, these data suggest that the chaperone protein HSP70 may control the turnover of PLXND1, and HSP70 inhibition may indirectly target PLXND1 in NEPC.

## Discussion

Trans-differentiation of prostate adenocarcinoma to acquire a neuroendocrine phenotype has been extensively investigated as a crucial mechanism for the development of NEPC, a highly aggressive and therapy-resistant phenotype of prostate cancer [22, 25, 33]. Emerging studies suggest that genes commonly upregulated in patients with a neuroendocrine phenotype regulate important neuronal functions and are associated with poor prognosis during cancer progression [22, 31, 32]. In this study, we identified PLXND1 as a target of NEPC, uncovering a PLXND1-dependent mechanism that induces a lineage switch of prostate adenocarcinoma cells towards a neuroendocrine phenotype. Prior research has indicated that the invasiveness and metastasis of AR-negative prostate cancer cells (PC3 and DU145) are regulated through Notch signaling, which transcriptionally regulates PLXND1[16]. Our results indicate that PLXND1 is more highly expressed in NEPC tumors than in prostate adenocarcinoma.

We demonstrated that enzalutamide treatment induced the emergence of a neuroendocrine phenotype, including small-cell histology and positive neuroendocrine markers, consistent with published evidence [12, 25, 34, 35]. Additionally, enzalutamide therapy was found to drive the upregulation of PLXND1 in C4–2B cells treated with enzalutamide for two months. To date, there have been no published studies investigating the correlation between the upregulation of PLXND1 and the emergence of a neuroendocrine phenotype in prostate cancer treated with enzalutamide. Further analysis indicated that the expression of PLXND1 is positively correlated with classic NEPC markers such as CHGA, NSE, SYP, and CD56, and is negatively correlated with AR and its targeting genes such as KLK2, KLK3, and NKX3–1. Recent integrated bioinformatic analyses of NEPC have revealed that the expression of neurolineage genes is positively associated with NEPC markers and negatively associated with AR and its target genes [34]. During the transition from prostate adenocarcinoma to NEPC and the acquisition of the neuroendocrine phenotype, the disease gradually changes from AR-dependent to AR-independent and is accompanied by the upregulation of PLXND1 expression. Furthermore, we found that AR signaling negatively regulates the expression of PLXND1 in advanced prostate cancer.

PLXND1, a member of the transmembrane protein family of plexins, is essential for axonal guidance and vascular patterning [14, 36]. Studies on endothelial cells revealed that PLXND1 acts as a direct force sensor and collaborates with neuropilin-1 and VEGFR2 to form a mechano-complex that elicits robust and global mechanical signaling upstream of the junctional complex and integrins [37]. Another study indicated that SEMA3E-PLXND1 signaling is a critical determinant of synaptic connections in sensory-motor circuits, specific for functional and anatomical rewiring of monosynaptic connections [38]. Recent evidence indicates that disruption of the axon guidance pathway mediated by SEMA3D and PLXND1 may slow the progression of pancreatic ductal adenocarcinomas [39]. PLXND1 may play a key role in the process of attaining a neuroendocrine phenotype and in the progression of NEPC. Our study found that knockdown or knockout of PLXND1 inhibited the proliferation of NEPC cells and organoids and decreased the tumorigenicity of NEPC cells in vivo. PLXND1 is highly heterogeneous in different types of tumors, acting as a tumor promoter in colon cancer and ovarian endometrioid cancer, and as a tumor suppressor in breast cancer [40, 41].

Currently, there are limited clinical trials on therapies for patients [42]. Treatment of NEPC mainly relies on clinical trial efficacy data for small-cell lung cancer [42]. Platinum-based chemotherapies, used for small cell neuroendocrine tumors, have shown response rates in the range of 10%–50% [43, 44]. Taxane-based and platinum-based chemotherapies are used to treat non-small cell variants of NEPC [32, 44]. In a previous clinical trial, 41 patients with metastatic androgen-independent prostate cancer and elevated serum CHGA or NSE levels received cisplatin and docetaxel treatment for three weeks. The results showed that 33% of these patients exhibited partial or complete responses, marked by reduced serum neuroendocrine levels, with an overall survival ranging from 1 to 38 months, with a median survival of 12 months. Nevertheless, over 50% of the treated patients experience toxic side effects, such as septic shock, asthenia, and neuropathy, highlighting the limitations of current chemotherapy-based therapies for NEPC [45]. Our results suggest that the chaperone protein HSP70 regulates the expression of PLXND1 through protein degradation. HSP70 is a ubiquitous molecular chaperone that acts on a large variety of cellular protein folding and remodeling processes. It also contributes to key steps in protein degradation through the ubiquitin-proteasome system and various autophagy pathways [46, 47]. Previous data from our research showed that the HSP70/STUB1 complex controls the sensitivity to AR-targeted therapy in advanced prostate cancer by synergistically degrading AR and AR-V7 [48]. In this study, we found that HSP70 binds to PLXND1 and regulates protein folding and degradation. Using an HSP70 inhibitor may indirectly target the PLXND1 protein and disrupt its signaling axis to improve the therapy of patients with NEPC.

Further investigation is needed to determine whether HSP70 cooperates with other proteins to degrade PLXND1, and its specific mechanism. Additionally, previous studies have indicated that PLXND1 always binds to its ligand, SEMAs [49]. SEMA-PLXND1 signaling plays important roles in cardiovascular, nervous, and immune system development as well as in cancer biology [39, 41, 50]. One potential research direction is to explore whether PLXND1 cooperates with the SEMA family members to regulate disease progression in NEPC.

In summary, our research uncovered an important process in which PLXND1 plays a key role in gaining neuroendocrine phenotypes in prostate cancer cells, which is a critical factor in the progression of NEPC. We suggest that targeting PLXND1 may be a promising strategy for treating NEPC. This suggests that efforts directed towards understanding and manipulating the PLXND1-driven mechanism could potentially lead to effective strategies for managing NEPC, addressing a critical need for prostate cancer therapeutics.
